# A qualitative study on the background of long-term maintenance patients at a private Japanese dental clinic

**DOI:** 10.1186/s12903-016-0203-2

**Published:** 2016-04-01

**Authors:** Tomotaka Kato, Seiichi Sugiyama, Michiko Makino, Toru Naito

**Affiliations:** Section of Geriatric Dentistry, Department of General Dentistry, Fukuoka Dental College, Postal address: 2-15-1, Tamura, Sawara-ku, Fukuoka-city, Japan; Sugiyama Dental Clinic, 1-53 Murakami-danchi, Yachiyo-city, Japan

**Keywords:** Maintenance, Patient background, Qualitative study

## Abstract

**Background:**

Continued periodontal maintenance after active therapy is highly important for maintaining a healthy oral function. In Japan, the rate of consultation for periodontal maintenance is remarkably low compared to other developed countries. This study analyzed the relationship between long-term maintenance and patient background characteristics in an effort to identify ways to increase the rate of consultation for periodontal maintenance in Japan.

**Methods:**

Thirty-three long-term maintenance patients were interviewed. The interviews were recorded on video. The conversation between the patient and the interviewer was converted to text, and the data were qualitatively analyzed using the Steps for Coding and Theorization (SCAT) method.

**Results:**

The mean age of the patients was 61.4 years and the average duration of maintenance was 10.7 years. The majority (90.9 %) of patients cared about their dietary habits, and 72.7 % of the patients understood the importance of physical activity. All of the patients wished to continue the maintenance, and 72.7 % of patients had good feelings about the staff of the dental clinic. However, their recognition of the description of primary prevention was low, with a response rate of only 21.2 %.

**Conclusions:**

The long-term maintenance patients had a high level of consciousness regarding their health and good feelings about the staff of the dental clinic. Oral hygienists, who are the main staff involved in periodontal maintenance were suggested to be important for increasing the maintenance consultation rate.

## Background

Japan has become a super-aged society, and the increasing burden of medical expense is a serious issue. To ease the burden associated with this issue, the primary prevention of lifestyle diseases, including diabetes mellitus and hypertension is of great importance [1, 2]. In the dental field, the primary prevention of dental caries and periodontal disease is highly important. Periodontal maintenance is necessary for the prevention of periodontal disease. Periodontal maintenance involves the monitoring of patients after active periodontal therapy in order to prevent reinfection and the continued progression of periodontal disease [3]. Axelsson et al. reported that periodontal maintenance was associated with a decrease in tooth mortality [4]. Periodontal maintenance involves the monitoring of individual patients and is influenced by systemic and genetic conditions, cigarette smoking, and the age of the patient [5]. A significantly higher incidence of periodontal disease is seen in patients who do not undergo periodontal maintenance therapy in comparison to those who regularly visit a clinic to undergo periodontal maintenance (odds ratio, 3.2) [6]. The absence of periodontal maintenance was equal to smoking as a risk factor for periodontal disease [6]. In addition, periodontal maintenance was shown to be the only way to maintain the effects of periodontal treatment [7]. In recent years, the recognition of the importance of periodontal maintenance has been growing in Japan [8]. There is no established method for calculating the rate of consultation for periodontal maintenance. However, it is clear that rate of consultation for periodontal maintenance is very low in Japan and the Japan Medical Data Index indicates that dental patients are frequently retreated for issues of dental health [9, 10]. These factors indicate the need to increase the rate of consultation for periodontal maintenance. However, there have been few studies on the background of patients who regularly undergo periodontal maintenance [11]. With regard to studies that have been performed in Japan, no studies have directly addressed patient background in relation to periodontal maintenance, while few studies have examined the causative factors of tooth loss during periodontal maintenance [12]. The lack of published studies on patient backgrounds in relation to periodontal maintenance may be due to the limitations associated with quantitative studies, which may not completely explain a patient’s real feelings or the essential information about their background. Thus, to investigate patient background, it is clear that a qualitative study would be more effective [13]. The Step for Coding and Theorization (SCAT) is an easily accessible qualitative data analysis method [14]. SCAT has recently been used in qualitative studies in medicine and dentistry [15, 16]. The aim of this study was to analyze the relationship between long-term maintenance and patient background factors using SCAT in an effort to the rate of consultation for periodontal maintenance could be increased in Japan.

## Methods

### Ethical considerations

Permission for this study was obtained from the Ethics Committee for Clinical Research at Fukuoka Dental College (approval number 183). To maintain patient anonymity, the personal information relating to the patients of the study was kept at the private clinic, and their names were replaced by ID numbers. Patient anonymity was ensured because the analysts did not have to access any identifying information.

### Patients

This qualitative study was conducted at one general dental clinic in Japan’s Chiba prefecture. The inclusion criteria were as follows: patients who had visited the clinic for periodontal maintenance every other month covered under the Japanese national health insurance system for more than 7 years and who had visited for maintenance on more than ten occasions. The patients were sent a letter of explanation about the study and a request for consent. The study was explained again when they visited the clinic. In total, 34 patients met the inclusion criteria and consented to participate in the study.

### Interview process

The study participants underwent a one-on-one interview at the clinic when they visited for maintenance. Interviewers received semi-structured interview training for calibration. They were not members of the clinic’s staff. The interview consisted of questions on the following information categories: “System health consciousness,” “Oral health consciousness,” “Reasons for continued maintenance”, “Hobbies or interests,” and “Life situation,” with open-ended questionnaire responses. A video recording was made of the interview with the consent of the patients. The recorded conversation between the patient and interviewer was then converted to text. The interviews were conducted within a period of 20 min so as to avoid adverse consequences in the patient’s periodontal maintenance.

### Data analysis

The text data of the interview was analyzed qualitatively by the “Steps for Coding and Theorization” method (SCAT). This method is a sequential and thematic qualitative analysis technique. It consists in the following 4 steps.

1. Focused words from within the interview text.
2. Words outside of the text which are replaceable with the words from 1.
3. Words which explain the words in 1 and 2.
4. Themes and constructs, including the process of writing a story-line and offering theories that weave together the themes and constructs.

As stated above, itemized theories were extracted from each of the patients. Among the patients, similar itemized theories were merged and percentages were shown for all patients.

## Results

1. Patient demographics
  A total of 34 patients were interviewed. One patient was excluded from analysis, because his/her responses digressed from the focus of the interview. The demographics of the patients are shown Table 1: the average age of patients was 61.36, the average duration of the dentist-patient relationship was 16.12 years, the duration of continued maintenance was 10.72 years, and the average number of visits for periodontal maintenance was 23.09. The smoking status was determined according to the US Centers for Disease Control and Prevention (CDC) classification as follows: current smokers (*n* = 4), former smokers (*n* = 7), and non-smokers (*n* = 22). The average interview length was 9.76 min.

**Table 1:** Patient demographics

	Mean	SD
Age (year)	61.36	13.93
Gender (male/female)	14/19	
Duration of Dentist-Patient Relationship (year)	16.12	5.61
Duration of Continued maintenance (year)	10.72	2.43
Number of Visits for Dental Maintenance	23.09	8.31
Smoking status (Current/Former/Non-smoker)	4/7/22
2. SCAT analysis and data integration
  There were various itemized theories from the interview that were subjected to SCAT analysis. For instance, the itemized theories from Patient No. 1 were “Friend’s introduction,” “Good impression of the clinic’s staff,” “Dental fear,” “Understanding the importance of exercise,” “Understanding the importance of dietary habits,” “Preference for a clean clinic,” “No interest in BGM(back ground music),” “Visit dental clinic as a family,” and “Hope to carry on consultation.”
  The itemized theories were aggregated into the following three categories: “Patient background” was used to define the patient’s daily life or environment; “Relationship with the dental clinic” defined what the patients thought about their regular dental clinic, and “Patient understanding of periodontal maintenance”: defined what the patients thought about periodontal maintenance or dental treatment.
3. Patient background
  The itemized theories about “Patient background” are shown Fig. 1. The most common itemized theory was, “Understanding the importance of dietary habits.” At least 90.9 % of patients understood the importance of dietary habits. Regarding exercise, 72.7 % patients understood the importance of exercise, and 69.7 % patients engaged in exercise. The percentage of patients whose interviews indicated that they spent time on holidays being involved in volunteer activities and participating in hobbies (“Active lifestyle on holidays”) was 69.7 %. The percentage of patients who indicated that they have an acquaintance with systemic disease (“Have a sick acquaintance”) was 60.6 %.

**Fig. 1:**
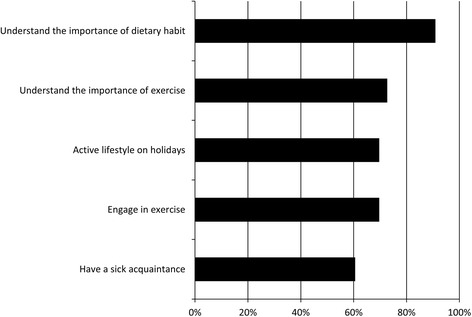
Patient background (the characterestics of the patient’s daily life or environment)
4. Relationship with the dental clinic
  From the contents of each patient’s interview, various itemized theories were recognized as corresponding to, “Relationship with the dental clinic.” The most common itemized theory was, “The hope of carrying on consultation,” which was indicated by all of the patients (Fig. 2). The second most commonly itemized theory was, “Good impression of clinic’s staff,” which was indicated by 75.9 % of the patients. Almost half of the patients (51.7 %) indicated that they “Visit the dental clinic as a family.” However, the number of patients that understood that recording incidents of periodontal pocket and bleeding on probing are important markers of periodontal disease (“Maintain patient history”) was only 41.4 %; while, “Reassuring environment” and “Good impression of PMTC” were both 21.2 %.

**Fig. 2:**
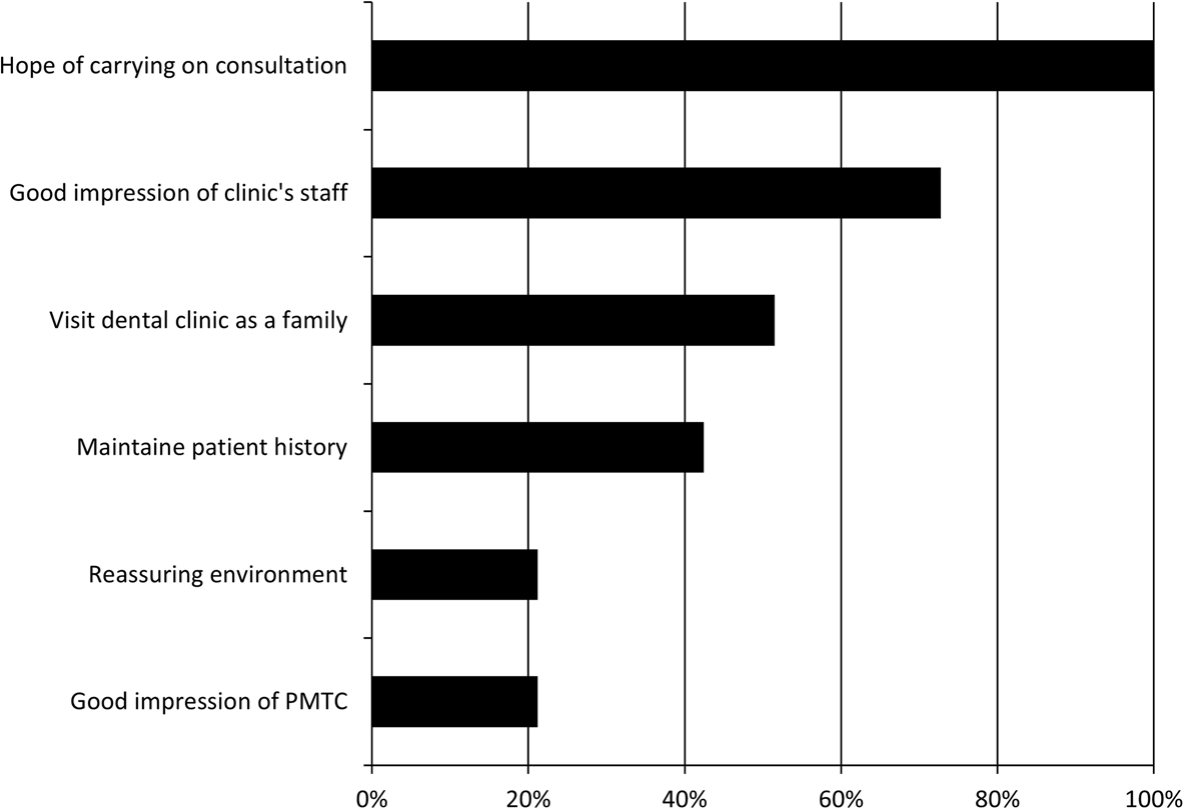
The relationship with the dental clinic (what the patients thought about their regular dental clinic)
5. Patient understanding of periodontal maintenance
  The patient’s relationship with dental treatment and periodontal maintenance, understanding of periodontal maintenance as indicated by the item,”Patient understanding of periodontal maintenance” is shown Fig. 3. The most common itemized theory was “Patient-modified behavior” (54.5 %). This theory contained such aspects as abstaining from smoking, improving dietary habits, refinement of dental self-care. The second most commonly itemized theory was, “Improvement of motivation” (42.4 %). This meant that visiting the dental clinic for maintenance improved the patient’s motivation with regard to oral hygiene. However, “Understand the importance of prevention,” was only indicated by 21.2 %. In contrast, there were many itemized theories on the expected effects of periodontal maintenance, such as, “Desire dental scaling” and, “Early detection, rapid cure.” “Dental fear” was only indicated by 6 % of patients.

**Fig. 3:**
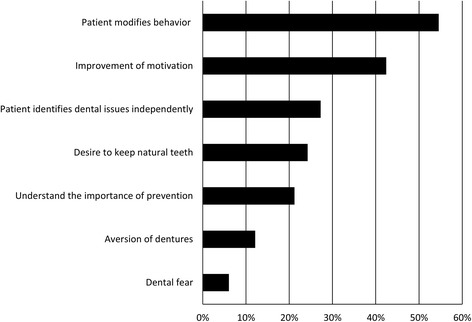
Patient understanding on periodontal maintenance (what the patients thought about periodontal maintenance or dental treatment)

## Discussion

In this study, we performed interviews with long-term maintenance patients at a Japanese private dental clinic, and analyzed their relationship with the dental clinic, their understanding of periodontal maintenance and their background.

According to “Patient’s background,” over 90 % patients reported that they were mindful in their dietary habits and over 70 % patients reported that they understood the importance of exercise. Therefore, it was indicated that the long-term maintenance patients had a high level of health consciousness. Furthermore, there were many indications of, “Active lifestyle on holidays,” and it was suggested that long-term maintenance patients had an active lifestyle. Because of this, it was thought that patients who had a high level of health consciousness and who lived an active lifestyle had a high possibility of undertaking long-term maintenance. Regarding the relationship with the dental clinic, all patients hoped to carry on consultation. Therefore, it was indicated that long-term maintenance patients would continue to undergo periodontal maintenance. Over 70 % patients had a good impression of the clinic’s staff, and almost all of the staff members who made a good impression with patients were dental hygienists. As a result, staff development is considered to be very important. Teraoka et al. also reported that periodontal maintenance patients had a high level of satisfaction with their dental hygienist [17]. Because of these findings, the cultivation of good communication skills in dental hygienists is considered to be a key to improving the rate of consultation for periodontal maintenance in Japan.

In this study, it was indicated that periodontal maintenance might improve a patient’s daily habits, because “Patient-modified behavior” was accounted for by over half of the respondents in, “Patient understanding of periodontal maintenance.” In addition, it was suggested that periodontal maintenance might contribute to the patient’s general health, since the content of “Patient-modified behavior” contained such items as abstaining from smoking and the improvement of dietary habits. However, only 20 % indicated “Understand the importance of prevention,” and there were many itemized theories such as, “Desire dental scaling,” and “Early detection, rapid cure.” Therefore, many patients might think that undertaking periodontal maintenance would make dental therapy easier. In Japan, Morishita also reported that very few maintenance patients understood the importance of prevention [18]. This study showed that only 196 of 712 maintenance patients (27.5 %) visited the clinic for the purpose of prevention of periodontal disease and dental caries.

Regarding the cause of the lower understanding in regard to prevention, it was thought that instruction on self-care was deficient. Moreover, it has been reported that that professional dental cleaning maintained the good condition of the oral environment [19]. However, Lang et al. reported that without the brushing of teeth, even dental hygienists made a plaque score of zero, and gingivitis developed after 48 h [20]. In addition, there are many reports showing that plaque control by patients is necessary and that patient motivation is important in this regard [21–23]. These points indicate the value of striving to keep patients informed about the importance of prevention.

This study recruited long-term maintenance patients. The management of their oral health was the role of dental hygienists. Furthermore, the patients reported that their reason for undergoing periodontal maintenance had more to do with the urging of their dental hygienist than the urging of their dentist, indicating the important role of dental hygienists. In this study, there were many itemized theories regarding a friendly relationship between patients and dental hygienists, and there were also many itemized theories on behavior modification in response to a dental hygienist’s instructions.

In the light of this study and these reports, it was considered that the instructions of dental hygienists contributed to the enhancement of the maintenance consultation rate.

This study was associated with some limitations. It was qualitative study, thus there were limitations in the analysis of the background of long-term maintenance patients. However, the findings from this study might be useful for future quantitative studies, which would require a clear understanding of the qualitative aspects of patient background.

## Conclusion

In the present study, patients who had a high level of consciousness regarding their health and lived an active lifestyle showed a high possibility of undertaking long-term maintenance. And, it was suggested that oral hygienists were very important to establish a long-term therapeutic relationship.

## Abbreviations

BGM: back ground music
PMTC: Professional Mechanical Tooth Cleaning
SCAT: Steps for Coding and Theorization
